# Supplementary material for: Multi‐dataset identification of innovative feature genes and molecular mechanisms in keratoconus
Source: J Cell Mol Med. 2024 Sep 19;28(18):e70079. doi: 10.1111/jcmm.70079 (PMC11412914; doi:10.1111/jcmm.70079)
Supplement: Supplementary file 2 — Material S2. [file JCMM-28-e70079-s002.zip › 7.GSEA analysis/4.MYRF/KEGG_HOMOLOGOUS_RECOMBINATION.html]

Details for gene set KEGG\_HOMOLOGOUS\_RECOMBINATION[GSEA]

|  || Dataset | MYRF.group.cls#h\_versus\_l |
| Phenotype | group.cls#h\_versus\_l |
| Upregulated in class | h |
| GeneSet | KEGG\_HOMOLOGOUS\_RECOMBINATION |
| Enrichment Score (ES) | 0.32017773 |
| Normalized Enrichment Score (NES) | 1.0587274 |
| Nominal p-value | 0.41586074 |
| FDR q-value | 1.0 |
| FWER p-Value | 0.999 |
Table: GSEA Results Summary

  

Fig 1: Enrichment plot: KEGG\_HOMOLOGOUS\_RECOMBINATION      
 Profile of the Running ES Score & Positions of GeneSet Members on the Rank Ordered List

  

| SYMBOL | TITLE | RANK IN GENE LIST | RANK METRIC SCORE | RUNNING ES | CORE ENRICHMENT || 1 | RPA4 | na | 616 | 0.541 | 0.1946 | Yes |
| 2 | XRCC2 | na | 1592 | 0.391 | 0.3202 | Yes |
| 3 | XRCC3 | na | 10650 | 0.084 | 0.0921 | No |
| 4 | TOP3A | na | 12336 | 0.059 | 0.0667 | No |
| 5 | SSBP1 | na | 13497 | 0.044 | 0.0505 | No |
| 6 | RAD54B | na | 14190 | 0.036 | 0.0445 | No |
| 7 | POLD1 | na | 14812 | 0.030 | 0.0382 | No |
| 8 | RPA1 | na | 16107 | 0.017 | 0.0075 | No |
| 9 | BLM | na | 16615 | 0.012 | -0.0024 | No |
| 10 | POLD4 | na | 17303 | 0.006 | -0.0199 | No |
| 11 | RAD51C | na | 18171 | -0.002 | -0.0440 | No |
| 12 | TOP3B | na | 18850 | -0.009 | -0.0601 | No |
| 13 | POLD2 | na | 20484 | -0.027 | -0.0964 | No |
| 14 | RAD50 | na | 20774 | -0.031 | -0.0927 | No |
| 15 | RPA2 | na | 20868 | -0.032 | -0.0828 | No |
| 16 | RAD52 | na | 22102 | -0.047 | -0.0999 | No |
| 17 | POLD3 | na | 22606 | -0.054 | -0.0933 | No |
| 18 | RAD54L | na | 22870 | -0.058 | -0.0783 | No |
| 19 | RPA3 | na | 23148 | -0.062 | -0.0619 | No |
| 20 | MUS81 | na | 24135 | -0.078 | -0.0596 | No |
| 21 | RAD51B | na | 24562 | -0.085 | -0.0385 | No |
| 22 | BRCA2 | na | 25524 | -0.103 | -0.0257 | No |
| 23 | RAD51D | na | 27259 | -0.141 | -0.0202 | No |
| 24 | NBN | na | 27921 | -0.158 | 0.0229 | No |
| 25 | EME1 | na | 28789 | -0.185 | 0.0705 | No |
| 26 | RAD51 | na | 30852 | -0.256 | 0.1117 | No |
Table: GSEA details [plain text format]

  

Fig 2: KEGG\_HOMOLOGOUS\_RECOMBINATION      
 Blue-Pink O' Gram in the Space of the Analyzed GeneSet

  

Fig 3: KEGG\_HOMOLOGOUS\_RECOMBINATION: Random ES distribution      
 Gene set null distribution of ES for **KEGG\_HOMOLOGOUS\_RECOMBINATION**
